# A preliminary report of an educational intervention in practice management

**DOI:** 10.1186/1472-6920-4-15

**Published:** 2004-09-20

**Authors:** Gerald E Crites, Richard J Schuster

**Affiliations:** 1 Department of Internal Medicine, Wright State University School of Medicine, 128 E. Apple Street, 2^nd ^Floor/Fred Weber Building, Dayton, OH, 45409-2902 USA; 2 Division of Health Systems Management, Department of Community Health, Wright State University School of Medicine, 3139 Research Park Blvd., Kettering Ohio 45420-401 USA

## Abstract

**Background:**

Practice management education continues to evolve, and little information exists regarding its curriculum design and effectiveness for resident education. We report the results of an exploratory study of a practice management curriculum for primary care residents.

**Methods:**

After performing a needs assessment with a group of primary care residents at Wright State University, we designed a monthly seminar series covering twelve practice management topics. The curriculum consisted of interactive lectures and practice-based application, whenever possible. We descriptively evaluated two cognitive components (practice management knowledge and skills) and the residents' evaluation of the curriculum.

**Results:**

The mean correct on the knowledge test for this group of residents was 74% (n = 12) and 91% (n = 12) before and after the curriculum, respectively. The mean scores for the practice management skill assessments were 2.62 before (n = 12), and 3.65 after (n = 12) the curriculum (modified Likert, 1 = strongly disagree, 5 = strongly agree). The residents rated the curriculum consistently high.

**Conclusions:**

This exploratory study suggests that this curriculum may be useful in developing knowledge and skills in practice management for primary care residents. This study suggests further research into evaluation of this curriculum may be informative for practice-based education.

## Background

Practice management education for residents has traditionally included training physicians in management issues related to the practice environment, including fiscal management, leadership skills, business and management skills, and managed care concepts [1]. Managed care concepts include ethics, communication skills, payment systems, population medicine, informatics and disease prevention. Although in existence since the 1970's, most practice management curricula have focused on managed care concepts, with little attention to the other skills [2-7]. In 2001, educators from Tuft's University wrote a report for curriculum development in the evolving practice environment [8]. This report, which was synthesized from nine component reports of national medical educational organizations, recommended future curriculum development beyond the traditional scope of managed care curriculum. It recommended redefining practice management as a curricular domain of fiscal, business, and practice system management skills distinct from traditional managed care topics [8]. The ACGME has recognized the need for residency training in the evolving practice environment, and has recommended training to include practice-based learning and improvement and systems-based practice [9]. The regression of traditional third-party managed care plans also implies an increased value and need for practice management skills [10]. Given this broad support of practice-based learning, physicians will need ongoing practice management and health systems education for the foreseeable future.

A few studies on practice management curricula exist for resident education, but much more information is needed on successful curriculum design and evaluation [11-13]. We describe, in detail, a pilot practice management curriculum design using the evolving curricular theme for a group of primary care residents. We also report its initial analysis on improving resident knowledge and skills, and describe the residents' evaluation of the curriculum.

## Methods

### Educational setting

We developed the curriculum for the University Medicine/Pediatrics Practice (UMP). This practice is a primary care-oriented, faculty-resident practice on the campus of Wright State University. Thirteen internal medicine/pediatrics residents, five general internist faculty, two internal medicine/pediatrics faculty, and one pediatrics faculty practice here. The practice is managed by Premier Healthnet, a 100 physician multi-site primary care group. Although UMP's mission includes addressing the needs of indigent patients in the Dayton area, it is modeled after a community-based, teaching practice model. Therefore, faculty and residents are expected to use effective practice management skills in their individual practices. A typical resident from our program enters a small (1–5 physician) community practice upon graduation and practices both internal medicine and pediatrics.

### Needs assessment

In 2001, two faculty members (GEC and RJS) at the Wright State University Departments of Medicine and Community Health were identified as lead faculty for this curricular project. Primary care faculty in the Departments of Pediatrics and Medicine tasked these two lead faculty members to development a practice management curriculum to reflect the evolving practice theme. By "evolving practice theme," we mean teaching practice management topics similar to the Tuft's curricular theme.

One lead faculty member (GEC) performed a needs assessment on the Internal Medicine/Pediatrics residents at UMP in the spring of 2001. The assessment used qualitative analysis via informal interviews of two senior residents, one of whom was chief resident. The interviews included open-ended questions on the need for practice management knowledge (example question: "What do you need to learn this year to help prepare you for running a community practice?"). The needs assessment also included an open feedback session with the residents after discussion of potential topics at the monthly resident education meeting (majority of residents present).

The results of the need assessment were uniform; the residents felt inadequately trained in practice management. The lead faculty concluded that these residents had some training in a few specific content areas (i.e., coding), but lacked an overall basic practice management knowledge or skill.

### Curriculum design

The lead faculty met again in mid-2001 to design the curriculum. The goals of the curriculum were to give the residents a basic understanding of practice management concepts and skills in the evolving practice environment. The lead faculty were free to select the most effective methods to meet their goals. They did face some challenges. They were given only 30-minute time slots each month and had 12 months to accomplish these goals. They also had to show some objective evidence of its effectiveness and have support of the residents at the end of the 12 months to continue the curricular project.

The design process resulted in a series of seminars covering 12 topics, listed in Table 1, with objectives. The seminars began in July 2001, and concluded in June 2002. The lead faculty assigned teachers to each seminar who were content experts, and included a medical biller, a nurse manager, a health systems researcher, two local HMO medical directors, a financial advisor, a risk manager, and a WSU junior faculty member. The assignment of seminar teachers is listed in Table 1, and one lead faculty member (RJS) led two sessions (referred to as the health systems researcher listed under Revenue Management and Accounts Payable Management in Table 1). Although the seminar teachers were free to utilize any method and media to meet their session objectives, they were encouraged to use as much interactive teaching approach as possible. The sessions were primarily in the form of teacher-centered discussions augmented primarily with handouts, overheads, and slides. The seminar teachers often supplied references and reference materials as tools for the residents in their daily practices. We encouraged ambulatory practice faculty throughout the year to discuss with residents, during resident ambulatory practices, application of principles learned in the seminar series.

**Table 1:** Schedule of topics (in bold), teacher assignments, and objectives for the practice management seminar series

**Topic, teacher**	**Objectives**
**Basic Coding**, Medical Biller	Introduction to the Fee Ticket E/M and PT Basics ICD-9 Basics
**Revenue Management**, Health Systems Researcher	Health System Overview Payment Systems How Physicians Get Paid
**Optimizing Coding to Enhance Reimbursement**, Medical Biller	Reimbursable Diagnoses in Primary Care Using Modifiers Procedures and Medication Coding
**Physician Personal Finance**, Financial Advisor	Financial Goals Financial Planning
**Insurance Systems and Payment Mechanisms**, HMO Director #1	Insurance Contracts IPAs and Collective Bargaining
**Dynamics of Group Practice**, HMO Director #2	Partnerships Structures: Solo, Small Group, Multi-specialty Practices Physician Leadership and Consensus Building
**Getting a Good Job**, WSU Faculty Member	Finding Positions and Writing CVs The Interview Process Contract Negotiations
**Accounts Receivable Management**, Medical Biller	The A/R Sheet Fiscal Targets Collections Management
**Accounts Payable Management**, Health Systems Researcher	Minimizing Expenses in Primary Care Economics of Running a Primary Care Practice
**Human Resources**, Nurse Manager	Staffing Needs Assessment Hiring/Firing/EEO Payroll & Benefits Conflict Resolution
**Risk Management**, Risk Manager	Minimizing Medico-legal Risk in Practice
**Regulatory Restrictions in Practice**, Nurse Manager	Understanding CLEA, OSHA, and HIPPA

### Curriculum evaluation

The respondents were a convenience sample of Internal Medicine/Pediatrics residents from all four years of training. We used a pre-experimental (one-group pretest/posttest) design for this exploratory study. We descriptively evaluated the curriculum on two cognitive components: practice management knowledge and skills. We also assessed the residents' evaluation of the curriculum.

To evaluate practice management knowledge, we used a knowledge test consisting of identical 12 item (true/false statements), and each question covered one objective from each topical area from Table 1. One example of a test item in the content area of coding is: "An established patient who has an expanded problem focused history and exam may be billed at a 99215 level." We administered the 0-month test to the entire group immediately before the first seminar session. We administered the 12-month test to the entire group immediately after the last seminar session.

To evaluate practice management skills, we devised a survey of self-assessed practice management skills. The survey consisted of 12 statements, and each statement queried the residents to respond on their assessment of their own practice management skills. Each statement consisted of one specific skill from an objective from each topical area listed in Table 1. An example of one survey item in the content area of coding is: "I understand how to use modifiers with E/M (evaluation and management) coding." We based the responses to the statements on a modified Likert scale, with 1 being strongly disagree, and 5 being strongly agree. We administered the 0-month self-assessed skill survey to the entire group immediately before the first seminar session. We administered the 12-month self-assessed skill survey to the entire group immediately after the last seminar session.

To explore the residents' evaluation of the curriculum, we devised another survey. This survey consisted of four statements querying the residents on their overall assessment of this curriculum and practice management education in general. The statements from the survey are given in Table 2B. The responses were based on the same Likert scale described above. This survey was administered to the entire group immediately after the last session.

**Table 2:** Resident self-assessed practice management skills (A) and curriculum evaluation (B) (modified Likert scale: 1 = strongly disagree and, 5 = strongly agree)

**Evaluation component**	**0-month (n = 12)**	**12-month (n = 12)**
	**Mean (95%CI)**	**Mean (95%CI)**
**A: Self-assessed practice management skills:**		
Results from 12 item survey	2.62 (2.27 – 2.97)	3.65 (3.41–4.08)

**B: Evaluation of practice management curriculum:**	**Mean (1 SD)**	**Mean (1 SD)**

Practice management series was effective in teaching me basic practice management knowledge	NA	4.13 (0.61)
I feel more confident in my own practice skills because of this curriculum	NA	3.96 (0.45)
I feel practice management curriculum should be incorporated into primary care curriculum	NA	4.67 (0.65)
I would be interested in expanding my primary care curriculum to include more practice management education	NA	4.67 (0.49)

The process of test instrument development was the same for both the knowledge test and the self-assessed skills survey. One lead faculty member (GEC) would generate a list of candidate items based on each objective in Table 1. The second lead faculty member (RJS) would review the list and select and/or modify items to match the item content to the objectives listed. Thus, both instruments possessed good face validity. Reliability testing was not performed due to the small sample size. Post-hoc item analysis on the 0-month knowledge test showed that only 2 items were answered 100% correct and the lowest item scored was 33% correct for this group. This suggests minimal floor and ceiling effects in the item mix. All other items ranged from 52% to 92% correct.

## Results

The participants were the 13 Internal Medicine/Pediatrics residents, and represented all four years of training (2, 4^th^-year; 3, 3rd-year; 4, 2^nd ^year; and 4, 1^st^-year residents). A third year resident failed to complete the 0-month tests and surveys, and a first year resident failed to complete the 12-month tests and surveys. This left 12 responses for both sets (0- and 12-month) tests and surveys. The average attendance for the sessions was 12, with a range of 10–13 attendees.

**Figure 1:**
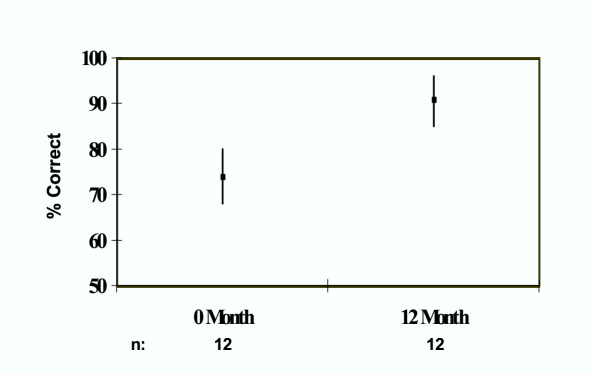
Practice knowledge test results (mean and 95% CI): before (0-month) and after (12-month) the course

The results from the knowledge test are given in Figure 1. As a group, the residents' mean score was 74% (95% CI, 68%–80%) for the 0-month survey and 91% (95% CI, 85–96%) for the 12-month survey. These confidence intervals do not overlap. This suggests that, if hypothesis testing were done, the results would probably reach statistical significance for the knowledge test.

On follow-up, we performed two post-hoc analyses. First, we were interested if these knowledge scores would decline over time. Therefore, we compared the knowledge test scores on the first six months topics to the scores on the last six months topics. Both sets of scores were derived from the 12-month knowledge test. We found that the mean scores appeared similar (first 6 months mean scores: 92% correct; the last 6 months mean scores: 90% correct).

Second, we were interested if the missing data on the 0-month and 12-month data could have impacted the results. Since one third-year resident completed the 12-month but not the 0-month test, we were interested in exploring if his responses on the 12-month test could have caused a larger difference between these two tests. After censoring his data, there appeared to be little impact on the 12-month results (censored mean score = 0.91, censored 95% CI, 85–96%). Additionally, the first year resident who failed to complete the 12-month test may have also impacted the results. Due to loss of identity links, we could identify her data to censor from the 0-month test. However, we censored the lowest score on the 0-month test as representing hers (this assumes that her score lowered the 0-month data the most, and, therefore, had the largest impact on 0-month mean score by skewing it away from the 12-month mean score). After censoring this data, we found no significant change in the 0-month results (censored mean score 0.75, censored 95% CI, 70–80%).

The results for the self-assessed skill survey are given in Table 2A. The mean scores on the 12-month survey (3.65) were higher than in the mean scores for the 0-month survey (2.62). The confidence intervals from this data do not overlap. This suggests that, if hypothesis testing were done, the results would probably reach statistical significance for the self-assessed practice management skills survey.

The results of the curriculum evaluation survey are given in Table 2B. All statements had a mean rating of greater than 3.90. The two statements assessing the residents' views towards practice management education in general (value of practice management education and the need to expand their education) both had mean scores of 4.67.

## Discussion

The practice environment continues to evolve [8]. Although a traditional term for educating physicians in the practice environment, "managed care curriculum" is a vague terminology and lacks comprehensiveness [8]. The Tufts' report did not use this term for specific curricular terminology, and this may parallel the purported demise of the term for the traditional payer system [10]. The Tufts' report included a comprehensive list of 10 curriculum domains in the evolving practice environment [8]. This report gave the practice management domain, which had lacked emphasis in half of its nine component reports, equal emphasis as the traditional managed care curricular domains [8]. The practice management domain included training on topics such as basic business skills, management skills, financial risk, payment systems, process improvement, and practice systems [8]. With respect to the evolving practice environment, the challenge for educators is devising practice management curricula that cover these topics adequately and relating them to other curricular domains (i.e., health systems, quality improvement, etc.).

We were interested in whether a curriculum design with this evolving theme may be useful in primary care education. We describe, in detail, a curriculum design similar to the evolving theme designed for a small group of primary care residents. The advantage of such a program as ours is its detailed design based on general and specific needs assessments and a description of evaluation methodologies. Our data suggests that this intervention may have had an impact on resident knowledge scores and self-assessed skills. Additionally, the residents appeared remarkably positive towards this practice management curriculum and practice management education in general.

A few studies have been published on practice management curricular design and evaluation for primary care residents. In a response to the growing need physician-managers, both Zoorob and Taylor and Johnson described curricular designs they proposed would fill this need [12,14]. Lynch and Johnson published a report on the evaluation of business management skills in primary care residents, and found no improvement with a short educational intervention (two day seminar) [11]. Werblun et al. described a proposed curriculum design and evaluation that would meet the needs for business management skills, and like our curriculum, recommended implementation over the course of the term of residency.

Our study does have some limitations. Because our small sample size, formal hypothesis testing was not possible and our data remains descriptive only. Stronger conclusions of these results would need to be re-evaluated with more subjects using formal hypothesis testing methods. Our experience suggests that internal motivation was probably one key factor to acceptance and apparent acceptance of this curriculum; the request for developing the curriculum came from our residents themselves. Also, the UMP faculty is uniformly positive towards developing these skills in themselves and in the residents, and this probably influenced residents' motivation to learn the subject matter. Since our faculty-resident practice is based on a primary care, community model, it may be difficult to generalize it to hospital-based practices or specialty residency training.

## Conclusions

We conclude that an extended curriculum in practice management with an evolving practice theme may be useful in primary care education. We also believe that attention to instructional design, including performing a needs assessments, using many teaching methods, and applying the concepts learned in learners' practices, may contribute to its acceptance and success. Future educational designs for this curriculum include its continued expansion, exploring more educational opportunities for implementation, and addressing specific characteristics of success and failure. Future educational research in this area would require a more formal research design to derive stronger conclusions regarding its effectiveness.

## Competing interests

None declared.

## Author contributions

GEC participated in the curricular needs assessments, curricular design, curriculum implementation, and drafting of the manuscript.

RJS participated in the curricular design, curriculum implementation, and drafting of the manuscript.

## Pre-publication history

The pre-publication history for this paper can be accessed here:


